# HIV-exposed uninfected infants: elevated cord blood Interleukin 8 (IL-8) is significantly associated with maternal HIV infection and systemic IL-8 in a Kenyan cohort

**DOI:** 10.1186/s40169-018-0206-5

**Published:** 2018-09-10

**Authors:** Barbara Lohman-Payne, Benjamin Gabriel, Sangshin Park, Dalton Wamalwa, Elizabeth Maleche-Obimbo, Carey Farquhar, Rose Kerubo Bosire, Grace John-Stewart

**Affiliations:** 1 Institute for Immunology and Informatics and Department of Cell and Molecular Biology, University of Rhode Island, Providence, RI 02903 USA; 2 Center for International Health Research, Rhode Island Hospital, Providence, RI 02903 USA; 3 Department of Pediatrics, The Warren Alpert Medical School of Brown University, Providence, RI 02903 USA; 4 Department of Paediatrics and Child Health, University of Nairobi, Nairobi, Kenya; 5 Departments of Global Health, University of Washington, Seattle, WA 98104 USA; 6 Departments of Medicine, University of Washington, Seattle, WA 98104 USA; 7 Departments of Epidemiology, University of Washington, Seattle, WA 98104 USA; 8 Department of Medical Epidemiology and Biostatistics, Karolinska Institute, Stockholm, Sweden; 9 Centre for Public Health Research, Kenya Medical Research Institute, Nairobi, Kenya; 10 Department of Pediatrics, University of Washington, Seattle, WA 98104 USA

**Keywords:** HEU, IL-8/CXCL8, MIP-1α, MIP-1β, IL-6, MIP-3α, GM-CSF, IL-10, CXCL11, Viral load, In utero HIV exposure, Structural equation models

## Abstract

**Background:**

In low and middle income countries, human immunodeficiency virus (HIV) exposed, uninfected (HEU) infants demonstrate higher morbidity and mortality than their unexposed counterparts. To determine possible immune correlates of this effect, we investigated the impact of in utero HIV exposure on the uninfected neonatal immune milieu and maternal factors mediating these abnormalities in a cohort of vaginally delivered mother-infants. Samples of delivery and cord blood plasma were selected from 22 Kenyan HIV-infected women and their HIV exposed uninfected (HEU) infants drawn from the pre-ARV era, while 19 Kenyan HIV-uninfected (HU) women and their infants were selected from a control cohort.

**Results:**

Compared to HU cord plasma, HEU cord plasma contained significantly higher levels of pro-inflammatory cytokines interleukins (IL)-6 and -8 (both p < 0.001) and significantly lower levels of CXC motif chemokine 11 (CXC11) (p < 0.001). Mediation analysis demonstrated that maternal HIV infection status was a significant determinant of infant IL-8 responses: HEU status was associated with a ninefold higher infant:mother (cord:delivery) plasma levels of IL-8 (p < 0.005), whereas maternal viral load was negatively associated with HEU IL-8 levels (p = 0.04) and not associated with HEU IL-6 levels.

**Conclusions:**

Exposure to maternal HIV infection drives an increase in prenatal IL-8 that is partially mediated by maternal cytokine levels. Differences between maternal and infant cytokine levels strongly suggest independent modulation in utero, consistent with prenatal immune activation. Elevated pro-inflammatory signals at birth may interfere with T cell responses at birth and subsequently influence immune maturation and the risk of morbidity and mortality in HEU infants.

## Background

Successful implementation of global programs for the prevention of mother-to-child transmission (PMTCT) of HIV has decreased perinatal HIV infections from 500,000 to 15,000 cases per year worldwide in the last decade (UNAIDS, 2015). However, it is now evident that this has created a large and expanding number of HIV exposed but uninfected (HEU) infants. These infants experience increased rates of morbidity and mortality compared with their HIV unexposed (HU) counterparts. A recent meta analysis of 22 studies from low and middle income countries showed a 70% increase in mortality among HEU during the first 2 years of life, and several individual studies report a 3- to fourfold increase in mortality over HU [1, 2].

The preponderance of current evidence suggests that the immune systems of HEU children differ in fundamental ways from HU children including changes in the numbers and proportions of leukocyte subsets present from the first months of life [3–5]. At birth cord blood T cells from HEU children can mount HIV-1 specific responses, indicating exposure to circulating maternal HIV antigen [6, 7]. In response to a range of stimuli, HEU cord blood mononuclear cells (CBMC) produce different cytokine profiles and reduced T cell helper responses compared to HU CBMC [8, 9]. However, there is controversy in the field as to the cause of the observed immune changes in HEU infants, the relative contribution of maternal health, and relative differences observed in HIV unexposed infants from the same communities. It is plausible that chronic in utero exposure to the maternal environment of persistent HIV infection affects the developing neonatal immune system, resulting in the immune alterations observed at birth. Equally plausible is that exposure to antiretroviral medications, poor nutrition, or exposure to multiple environmental antigens other than HIV, could stimulate a similar profile in HIV unexposed infants living in similar conditions.

Here we designed a retrospective study using careful specimen selection to systematically minimize the above factors known or suspected to confound interpretation of HEU data. Specifically we drew from specimen and data archives established prior to ARV interventions and purposefully selected mother-infants pairs with a clinical medical record indicating no infections or hospitalizations during the study duration in order to minimize the effects of environmental pathogens. Moreover we selected HEU infants and HU control infants of similar birthweight and gestational age and chose to evaluate infant cord blood as a window into prenatal immunity. Here we demonstrate cord blood plasma T cell cytokines and chemokines from HIV uninfected infants born to HIV infected women not taking ARV are markedly different from HIV unexposed infants. HIV positive maternal T cell cytokines were markedly lower than HIV uninfected women at delivery, and despite this, HEU cord plasma contained significantly elevated levels of the pro-inflammatory and chemotactic cytokines IL-6 and IL-8 and reduced levels of an interferon gamma (IFN-γ)-induced chemokine, CXCL11 compared to HU infants.

## Methods

### Cohort and specimen selection

This study used specimens from two cohort studies. Cohort 1 was a randomized controlled trial conducted 1992–1998 in Nairobi, Kenya prior to the availability of antiretrovirals for prevention of mother-to-child-transmission, as previously described [10]. HIV seropositive women were enrolled during pregnancy, and provided written informed consent for participation in the study and storage of specimens. Maternal peripheral blood was collected when women presented at the hospital at onset of delivery. Maternal plasma HIV-1 RNA levels at delivery were determined using a transcription-mediated amplification (TMA) method sensitive for the detection of multiple HIV subtypes (Gen-probe HIV-1 viral load assay) [11]. We selected samples from the HIV infected cohort based on proportional representation across quartiles of maternal HIV viral load, as a surrogate for levels of fetal exposure to HIV. Infants were examined and cord blood was collected at birth. Infants were tested quarterly for HIV-1 using HIV-1 PCR filter paper assays [12]. All infants selected for the current study were HIV-1 antibody negative at 24 months of age.

Cohort 2 enrolled HIV uninfected women between 2004 and 2006, as previously described [13]. The infection status of women enrolled in the HIV uninfected cohort was verified by two HIV-1 rapid tests (determine HIV-1/HIV-2 antibody/HIV-1 antigen test, Abbott Laboratories, Abbott Park, IL and Unigold HIV-1 antibody test, Trinity Biotech, Bray, Ireland). Women were recruited during pregnancy and provided written informed consent for participation and storage of specimens. Maternal peripheral blood was collected at delivery and infants were examined and cord blood collected at birth.

Women/infant pairs from both cohorts were selected to reduce factors suspected of influencing infant immunity: lack of history of sexually transmitted infection (STI), not currently receiving treatment for an STI, HIV uninfected singleton infant, normal birth-weight > 2500 g, and normal gestational age > 37 weeks, as defined in the parent study [10]. All specimen collection was conducted within the guidelines of the Declaration of Helsinki and all components of this study were approved by the Kenyatta National Hospital Ethics and Research Committee and the Institutional Review Boards (IRB) of the University of Washington and the University of Rhode Island.

### Detection of plasma cytokines

Peripheral and cord blood plasma was separated from EDTA-anticoagulated blood at the time of delivery and stored frozen at − 80 °C. We analyzed duplicate plasma samples for levels of interleukins (IL)-1β, IL-2, IL-4, IL-5, IL-6, IL-7, IL-8 (also known as CXCL8), IL-10, IL-12(p70), IL-13, IL-17A, IL-21 and IL-23, interferon (IFN)-γ, tumor necrosis factor (TNF)-α, granulocyte/macrophage-colony stimulatory factor (GM-CSF), as well as chemokines CCL3, CCL4, CCL20, CXCL11 and CX3CL1 using a multiplexed high sensitivity human T-cell cytokine magnetic bead panel (EMD Millipore Corp., Billerica, MA, USA). Three independent plates were run with standard- and plate-controls; women-infant pairs were grouped per plate. Assays were conducted in accordance with the manufacturer’s instructions. All cytokine values below the lower limit of detection were recoded as the lower limit of detection of individual cytokine standards.

### Statistical analysis

Cytokine data were tested for outliers and reported as medians and interquartile ranges. Medians of related samples were compared using Wilcoxon rank sum test and independent sample medians were compared using Mann–Whitney U. The cohort size for paired samples of HIV-infected women at delivery and their infant cord blood was 22, while the cohort size for HIV uninfected women at delivery and their infant cord blood was 19. In unpaired analyses, data from an additional 8 HEU cord blood specimens were included. Linear regression was used for analysis of cord blood IL-8 and viral load. Bonferroni correction for multiple comparisons was applied to multiplexed cytokine data.

Mediation analyses using structural equation modeling (SEM) [14, 15] were conducted to determine whether all, or part, of the relationship between maternal HIV status (infected/uninfected) and cord blood cytokine levels was explained by maternal cytokine levels. Mediation analyses were also performed to determine whether maternal cytokine levels mediated the relationship between maternal viral load and infant cord blood cytokines. Data were natural log transformed for mediation analysis. Statistical analyses were performed using Prism (version 6.0f, GraphPad, La Jolla, CA) and SAS 9.4 software (SAS Institute, Cary, NC). A p value < 0.05 was considered to be statistically significant, unless corrected for multiple comparisons.

## Results

### Cohort description

The HIV-1-infected women selected for this sub-study were representative of the original HIV cohort as young, moderately immunosuppressed, without a medical history of HIV-related illnesses. Specifically, the median age at enrolment was 25 years (interquartile range (IQR), 23–28). The women had a median CD4+ T cell count of 403 cells/ml (IQR, 313–523) and CD4:CD8 ratio of 0.44 (IQR, 0.29–0.73). Nineteen percent of the HIV positive women had a CD4:CD8 ratio greater than 1.0. The median viral load at delivery was 4.6 log_10_ RNA copies/ml plasma (IQR, 4.4–5.1). The HIV uninfected mother-infant cohort was enrolled from a similar community. The median age at enrolment was 25 years (IQR, 23–29). Infants born to HIV infected and HIV uninfected groups of women were of similar birth-weight: 3.3 kg (IQR, 3.0–3.6) vs 3.1 kg (IQR 2.9–3.5), respectively.

### HIV-infected women have increased IL-8 at delivery compared to HIV-uninfected women

The infant’s nascent leukocyte responses occur in the presence of ‘pre-conditioned’ prenatal cytokine milieu, based on maternal–fetal exposures. We chose to evaluate maternal delivery specimens as a reflection of prenatal and early life exposures. All pregnant women regardless of HIV status presented at delivery with detectable pregnancy-supporting cytokines IL-4 and -10, although the HIV-infected women had significantly lower levels of IL-4 (14 pg/ml (IQR, 8–19) vs 136 pg/ml (IQR, 84–164) and IL-10 (5 pg/ml (IQR, 4–9 vs 19 pg/ml IQR, 8–23, p < 0.001, respectively), compared to HIV-uninfected women (Table 1).

**Table 1:** Maternal plasma mediators at delivery from HIV-uninfected and HIV-infected women

Mediator, pg/ml	LLD	HIV uninfected n^a^ = 19	HIV infected n = 22	p value^b^
		Median (IQR)	Median (IQR)	
Pro-inflammatory				
IL-23	8.0	194 (151–408)	53 (29–103)	< 0.001
IL-17α	0.7	7 (5–12)	2 (1–4)	< 0.001
IL-12(p70)	0.5	5 (4–8)	2 (1–2)	< 0.001
TNF-α	0.4	8 (7–10)	6 (5–7)	< 0.001
IL-1β	0.5	5 (5–7)	2 (1–5)	0.25
IL-6	0.2	9 (6–30)	6 (2–40)	0.25
Chemotactic				
IL-8/CXCL8	0.3	9 (8–13)	36 (15–361)	< 0.001
CX3CL1	18.0	333 (205–459)	71 (48–120)	< 0.001
CCL20	0.6	22 (19–27)	15 (13–20)	< 0.002
CXCL11	1.5	157 (93–236)	45 (24–85)	< 0.001
CCL3	0.3	30 (25–34)	13 (10–57)	0.25
CCL4	1.0	33 (27–46)	13 (7–56)	0.02
Immunoregulatory				
IFN-γ	0.6	21 (17–32)	12 (11–17)	< 0.001
IL-7	0.4	14 (10–18)	7 (5–10)	< 0.001
IL-2	0.5	0 (0–5)	2 (1–3)	0.09
IL-21	0.3	3 (1–5)	2 (1–4)	1.0
Th2				
IL-4	1.8	136 (84–162)	14 (8-19)	< 0.001
IL-5	0.5	4 (2–5)	2 (1-2)	< 0.001
IL-10	1.5	19 (8–23)	5 (4-9)	< 0.001
GM-CSF	1.2	282 (132–358)	37 (21-51)	< 0.001
IL-13	0.3	10 (5–16)	5 (4-8)	0.01

Bonferroni correction for multiple comparisons was applied; thus p < 0.003 was considered significant

*LLD* lower limit of test kit detection, pg/ml

^a^Number women with plasma samples available at time of delivery

^b^Independent sample medians were compared using Mann–Whitney U test

Unsurprising for a population of women with chronic untreated HIV and a median CD4 of ~ 400 cells/ml, the HIV-infected women had significantly lower levels of 14 of the 21 T cells cytokines across the broad range of functions including pro-inflammatory, chemotactic, immunoregulatory, and Th2 type cytokines compared to HIV uninfected women. Interestingly, IL-8 was detected at a significantly higher level compared to HIV-uninfected women; HIV-infected women had a fourfold increase in levels of plasma cytokine IL-8 at delivery compared to uninfected women (36 pg/ml (IQR 15–361) vs 9 pg/ml (IQR 8–13) p < 0.001, respectively).

### Infants differing by HIV exposure status in utero: HEU infants are born with significantly increased levels of IL-6 and -8 and decreased levels of CXCL11 compared to HU infants

Of 21 T cell cytokines/chemokines measured, 15 were present at median concentrations above the lowest limit of detection (Table 2). Pro-inflammatory cytokines IL-6 and -23 were significantly different in HEU vs HU cord blood samples. Median IL-6 levels were ~ 25-fold higher in HEU infants compared to HU infants (24 pg/ml (IQR 2–155), vs 1 pg/ml (IQR 1–6), respectively). Levels of IL-23 were undetectable in the HEU infant cord blood; a subset of HU infant cord blood samples had detectable levels sufficient for the group medians to be significantly different (p < 0.001). Chemotactic cytokines IL-8 and CXCL11 were significantly different in cord blood plasma from infants exposed to HIV in utero (both p < 0.001). Median cord blood plasma IL-8 levels were > 50-fold higher in HEU infants compared to HU infants.

**Table 2:** Infant cord blood plasma mediators from HIV-Unexposed infants compared to HIV-exposed uninfected infants

Mediator, pg/ml	LLD	HIV unexposed (HU)		HIV exposed uninfected (HEU)		p^b^
		n^a^	Median (IQR)	n^a^	Median (IQR)	
Pro-inflammatory						
IL-6	0.2	17	1 (1–6)	29	24 (2–155)	0.001
IL-23	8.0	16	8 (8–16)	30	8 (8–8)	0.001
IL-1β	0.5	18	4 (1–39)	28	7 (0.5–102)	0.800
TNF-α	0.4	19	18 (15–26)	30	15 (9–38)	0.300
Chemotactic						
IL-8/CXCL8	0.3	19	13 (7–26)	29	748 (93–1882)	< 0.001
CXCL11	1.5	19	372 (279–428)	30	154 (65–254)	< 0.0001
CCL3	0.3	19	34 (17–70)	30	176 (29–1901)	0.030
CCL4	1.0	19	67 (61–121)	22	71 (290–152)	0.600
CCL20	0.6	19	40 (27–57)	26	47 (32–133)	0.100
CX3CL1	18.0	17	18 (18–28)	30	18 (18–18)	0.006
Immunoregulatory						
IL-21	0.2	15	0.2 (0.2–0.2)	30	0.7 (0.3–2.2)	0.001
IL-7	0.4	19	6 (2–13)	30	2 (0.4–4)	0.003
Th2						
IL-5	0.5	17	0.6 (0.5–0.9)	30	0.5 (0.5–0.5)	< 0.0001
IL-10	1.5	16	1.5 (1.5–7)	29	3 (1.5–6)	0.100
GM-CSF	1.2	19	7 (3–13)	30	5 (1.2–17)	0.700

The following cytokines were below the assay limit of detection in both populations: IFNγ, IL-12(p70), IL-13, IL-17α, IL-2 and IL-4

*LLD* lower limit of test kit detection, pg/ml

^a^Number samples with valid results

^b^Bonferroni correction for multiple comparisons was applied; p < 0.003 was considered significant

(748 pg/ml (IQR, 93–1882) vs. 13 pg/ml (IQR 7–26), respectively). CXCL11 was present at

significantly lower levels in HEU cord blood plasma compared to HU cord plasma (p < 0.001). CXCL11, also known as Interferon-inducible T-cell alpha chemotactic (ITAC) and Interferon-γ-inducible protein 9 (IP9), was present at approximately half the levels found in HU infants (154 pg/ml (IQR 65–254) vs 372 pg/ml (IQR 279–428)), while well above the lower limit of detection of 1.5 pg/ml). Immunoregulatory cytokine IL-21 and Th2 cytokine IL-5 levels were significantly different in cord blood plasma from HEU infants compared to HU infants although of note many individual’s IL-21 and IL-5 cord blood levels were at or near the limit of detection. The remaining cytokines tested were not significantly different between HEU and HU (IL-7, CX3CL1, CCL3, GM-CSF, IL-10, CCL20, IL-1β, CCL4, TNF-α) or were below the lower limit of detection in both populations (IFNγ, IL-12(p70), IL-13, IL-17α, IL-2 and IL-4).

### Cord blood plasma IL-8 and CXCL11 levels are increased compared to maternal delivery plasma

**Fig. 1:**
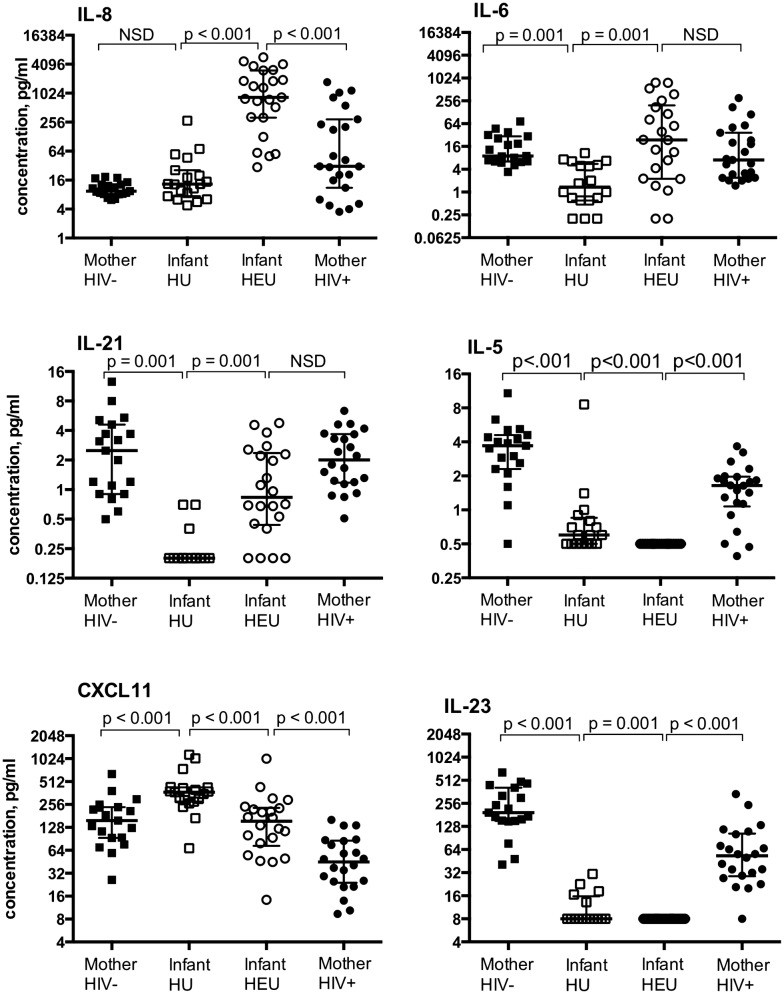
HIV exposed uninfected infant cord blood displays elevated cytokines consistent with inflammation and immune stimulation compared to HIV unexposed infant cord blood. Cytokines were detected in samples from all maternal delivery plasma and infant cord blood plasma using multiplexed Luminex Elisa bead-based technology. Assays at or below the lower limit of detection were assigned the lowest limit of detection. Individual plasma concentration (*ln* pg/ml), group median and IQR are plotted. Related sample medians (HEU vs HIV+ mother, n = 23 pairs; HU vs HIV− mother, n = 19 pairs) were compared using Wilcoxon Rank Sum test while independent medians were compared using Mann–Whitney U test (HEU vs HU). Symbols are as follows: *HIV− women* filled squares, *HU infants* open squares, *HEU infants* open circles, *HIV+ women* filled circles

We conducted paired analyses of cytokine levels present in cord plasma relative to levels in maternal plasma collected at delivery for the 23 HEU and 19 HU mother-infant dyads (Fig. 1). Median (IQR) HEU cord blood IL-8 concentration was significantly higher than that measured in maternal delivery blood plasma (748 pg/ml, (IQR 93–1882) vs 36 pg/ml (IQR 15–361); p < 0.001), suggestive of synthesis within the infant circulation rather than passive transfer across the placenta. Median (IQR) HEU cord IL-6 levels were not significantly higher than HIV + maternal blood plasma levels (24 pg/ml (IQR 2–155) vs 6 pg/ml (IQR 2–40), p = 0.019), while HU infants had lower cord blood IL-6 concentrations than their HIV uninfected mothers (1 pg/ml (IQR 1–6) vs 9 pg/ml (IQR 6–30), p = 0.001). Similarly, median (IQR) HEU cord IL-21 was lower and levels did not reach significance when compared to paired maternal delivery samples (0.7 pg/ml (0.4–2.2) vs 2.0 pg/ml (1.1–3.6); p = 0.014). A different pattern was observed for Th2 cytokine IL-5, which was largely undetectable in cord blood plasma collected from HEU infants and present at significantly lower levels compared with cord blood from HU infants (0.5 pg/ml, (0.5–0.5) vs (0.6 pg/ml (0.5–0.9), respectively).

The lower levels of CXCL11 and IL-23 observed in HEU relative to HU cord blood plasma were not due to lower maternal levels at delivery. Similar to our observation with IL-8, median (IQR) HEU cord blood levels of CXCL11 were significantly higher than maternal delivery blood plasma (153 pg/ml (IQR 65–254) vs 45 pg/ml (IQR 24–85) p < 0.001). In contrast, all individual HEU cord blood levels of IL-23 were below the limit of detection, while readily detectable in HIV + maternal plasma (53 pg/ml, (IQR 29–103). The data demonstrate cord blood mediators are not uniformly concentrated or diluted in infant cord blood, supporting a biologic mechanism mediating the observed difference in HEU cord blood as compared to a change in maternal–fetal vascular permeability.

### HIV exposure significantly alters pro-inflammatory cytokines present in cord blood compared to delivery blood

The process of delivery is dynamic and to reflect the physiologic range that exists for cytokines present in maternal and infant circulations at delivery and birth we determined a group median index from individual mother-infant pairs. An index > 1 indicates the cytokine level in cord blood exceeds the level measured in the infant’s mother’s delivery plasma.

**Fig. 2:**
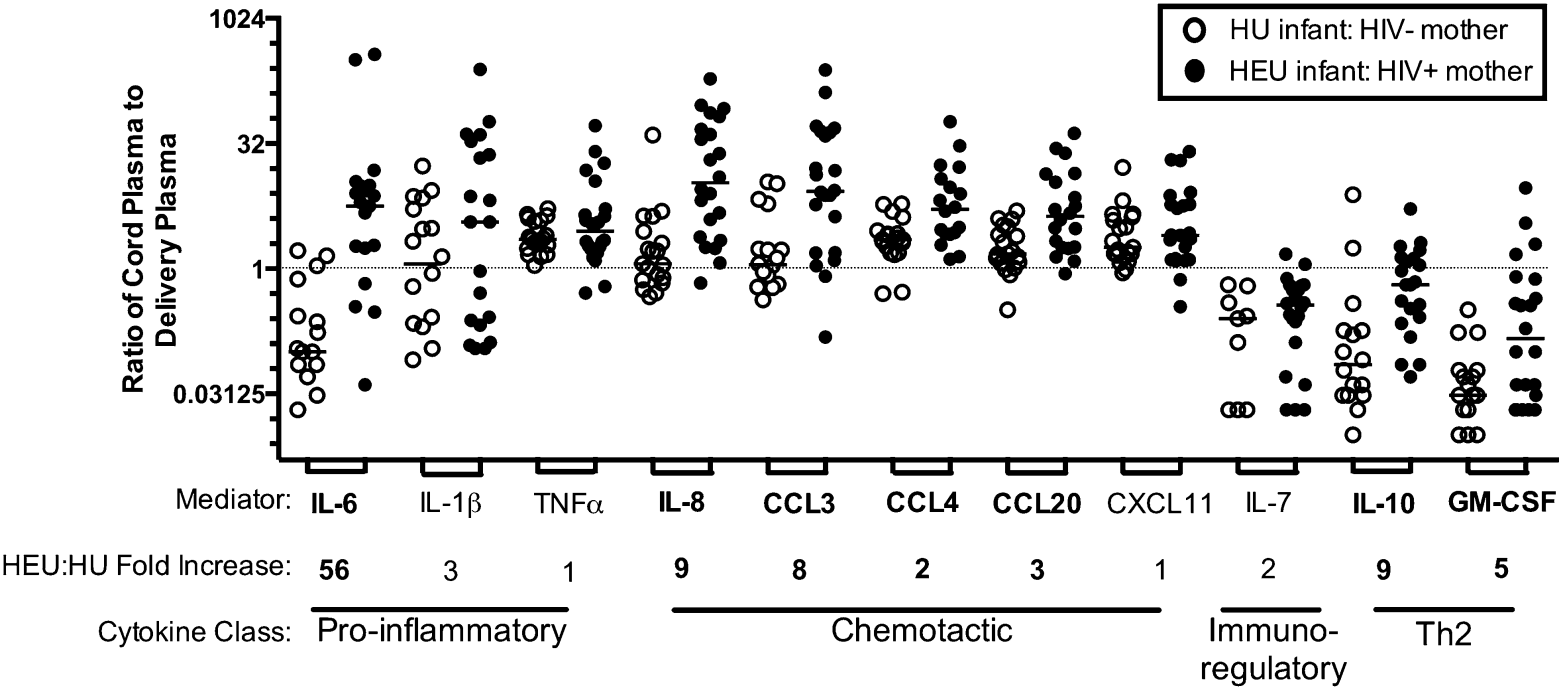
HIV infection associated with changes in delivery and birth cytokine patterns. Cytokines were detected in samples from all maternal delivery plasma and infant cord blood plasma using multiplexed Luminex Elisa bead-based technology and grouped by broad category. Assays at or below the lower limit of detection were assigned the lowest limit of detection. Cytokines undetectable in the majority of HEU or HU samples were not included: IL-5, IL-21, IL-23 and CX3CL1. The ratio of infant cord blood to maternal delivery blood plasma cytokine levels was calculated. Each cytokine ratio is plotted for HU infant-HIV negative mother (open circles) and HEU infant-HIV-infected mother (closed circles) and the fold change from the HU group median is indicated. Independent group medians were compared using Mann–Whitney U test with correction for multiple comparisons p < 0.005. Significant mediators are bolded

HIV uninfected women and their unexposed infants tended to have an index at or < 1 meaning maternal cytokines levels in blood at delivery were greater or equal to cord blood levels for IL-6, IL-8, IL-10, CCL3, GM-CSF, IL-1β, CCL20 and IL-7 (Fig. 2). HEU cord blood/delivery cytokine indices were significantly greater than HU indices for 7 of 11 detectable cytokines: IL-6, IL-8, IL-10, CCL3, GM-CSF, CCL20, and CCL4 (closed vs open circles, p < 0.005). Several cytokines levels were not significantly different in cord blood relative to delivery blood in HIV-infected women/HEU infants compared to HIV-uninfected women and their HU infants: IL-1β, IL-7, TNFα, and CXCL11.

### Maternal HIV infection, but not viral load, drives infant cord blood IL-8 levels

**Fig. 3:**
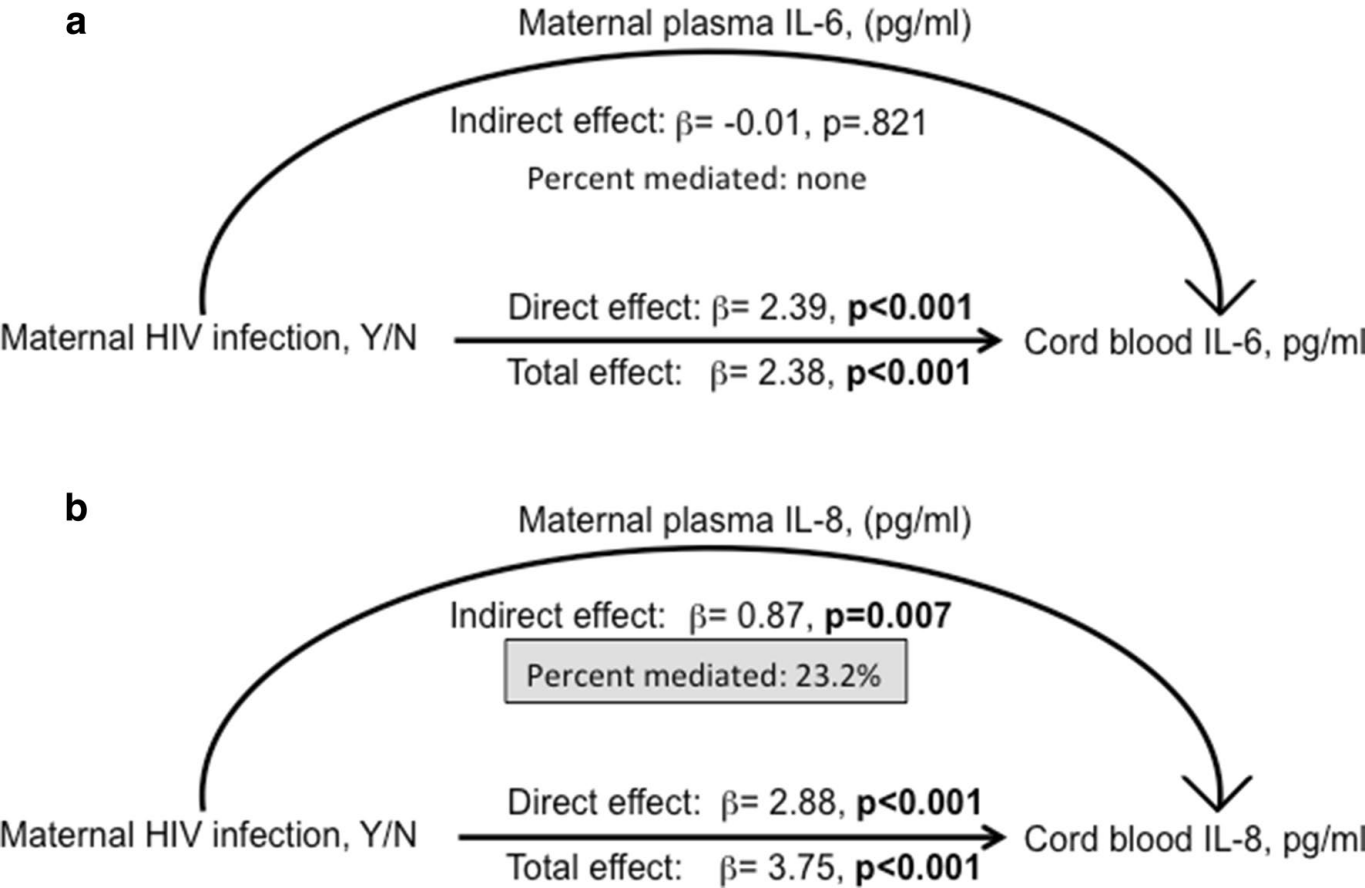
Maternal HIV infection drives infant IL-6 and IL-8 levels present at birth in HEU infants through different pathways. SEM models with maternal HIV infection (yes/no), maternal IL-8 levels at delivery in the pathway, and infant cord blood IL-8 levels as the outcome variable for 42 mother-infant pairs. HEU infants have 2.38 *ln* IL-6 levels in cord blood at birth and none of the infant IL-6 is attributable to maternal IL-6 levels (**a**). HEU infants have 3.75 times *ln* IL-8 levels in cord blood than detected in HU cord blood, and ~ 23% of this can be attributed to the maternal IL-8 levels (**b**)

Statistical modeling with structural equation mediation (SEM) is a powerful statistical approach to address interdependent relationships among multiple variables simultaneously and allows delineation of the relative importance of factors within the direct causal pathway on the outcome of interest [14, 15]. SEM was used to estimate the effect of maternal HIV infection working through maternal cytokine levels on infant cord blood cytokine levels. Maternal HIV infection status had a large significant total combined effect on both infant cord blood IL-6 and -8 levels (p < 0.001, Fig. 3). Maternal HIV infection did not mediate its effect on cord blood IL-6 through maternal plasma IL-6 levels (p = 0.8), indicating maternal HIV infection affects levels of infant cord blood IL-6 through pathways not measured in this study (Fig. 3a). However, maternal plasma IL-8 levels contributed 23% of cord blood IL-8 levels, a substantial percent for complex biological systems, while maternal HIV infection itself mediated 77% of the IL-8 present in cord blood through unmeasured pathways (Fig. 3b). Maternal HIV status had a significant but very small effect on cord blood IL-21; this cytokine was not included in subsequent models (total effect β = 0.02, p = 0.002).

**Fig. 4:**
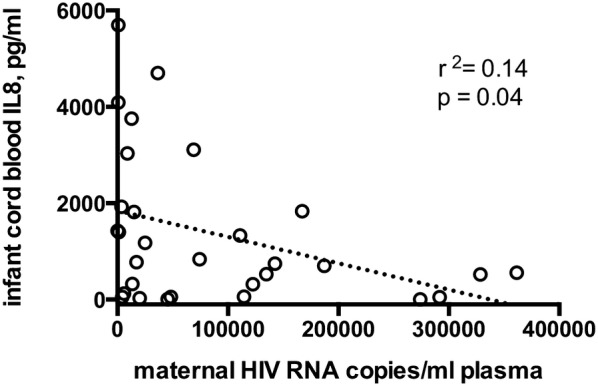
Linear regression of HEU infant cord blood IL-8 levels by maternal HIV at delivery. The impact of maternal HIV RNA copies/ml plasma on HEU plasma IL-8 concentrations was determined using linear regression. HIV RNA copies were determined using TMA method; IL-8 levels were determined using multiplexed ELISA format, both as described in methods. A negative linear association between maternal viral load and infant IL-8 concentrations at birth is shown by the dashed line, slope = − 0.0055

We hypothesized maternal viral load as a proxy for stimulation of prenatal pro-inflammatory cytokines directly. As shown in Fig. 4, the level of cord blood plasma IL-8 was negatively correlated with maternal viral load with a low correlation coefficient and a marginal p value (slope = − 0.006, r^2^ = 0.14, p = 0.04). Additionally, SEM with maternal viral load and IL-8 in the pathway for mediation of infant cord blood IL-8, revealed no significant effect of maternal viral load. Taken together these data do not support the hypothesis that maternal viral load directly stimulates infant IL-8 production; alternatively, the negative slope, weak r^2^ value and lack of significance in the SEM models indicate factors beyond viral load explain the pattern of HEU IL-8 levels at birth.

## Discussion

We hypothesized that maternal HIV, either directly via viral load/viral antigens or indirectly via altered plasma cytokines, would set a different immune stage for HEU infants than for HU infants. IL-6 and IL-8 levels present in cord blood of HEU infants were significantly elevated compared both to their HIV-infected mothers and to their HIV-unexposed community peers. Our study provides a direct link between maternal HIV infection and elevated levels of prenatal IL-6 and IL-8 measured in cord blood from infants who were born HIV uninfected and who did not acquire HIV through vaginal delivery and breastfeeding during the first year of life and who were unexposed to ARV. Our models demonstrate maternal IL-8 directly mediated ~ 25% of the effect of HIV infection on cord blood IL-8, with ~ 75% of the effect unmeasured in this study, indicative of alternative pathways or cellular sources of neonatal IL-8 induction and regulation. In contrast, maternal HIV infection did not mediate infant IL-6 levels via maternal IL-6, leaving all of the effect of maternal HIV infection on neonatal IL-6 induction unexplained in our models.

We have extended the finding that IL-8 is not limited to placental transfer in healthy women to that of HIV-infected-HEU mother-infant pairs [16, 17]. IL-8 is a major T cell effector in newborns and can be produced by endothelial cells, tissue macrophages, and cells of monocytic origin [18]. The IL-8 detected in cord blood here is plausibly derived from maternal or infant origin: from viral antigen-stimulated endothelial cells lining the umbilical cord, tissue macrophages present in the syncytiotrophoblast layer of the placenta or monocytic cells of infant ontogeny [19].

The onset of active labor and vaginal delivery is associated with elevated levels of IL-6 in both maternal and infant circulation [20], however we recorded a ~ 50-fold increase in the IL-6 index of cord blood to delivery blood observed in HEU compared to HU mother-infant pairs, the biggest difference amongst the mediators measured. Infection and inflammation during pregnancy are hypothesized to be associated with induction of placental cytokines, including IL-6, and increased fetal exposure to pro-inflammatory cytokines can result in increased risk of infant neurologic injury, even in the absence of HIV [21]. In addition to maternal infection/inflammation, direct transfer of microbes secondary to HIV infection may occur from the maternal bloodstream across the placenta into the fetal circulation, such as has been observed with increased gut microbial translocation [22]. Alternatively direct or indirect microbial LPS stimulation may induce placental cytokine production and the release or production of downstream mediators following Toll-like receptor signalling [23].

The elevated levels of IL-8 we observed in HEU but not in HU cord blood could result from a combination of increased production or decreased down-regulation of IL-8. As part of the pro-inflammatory antiviral immune response, intracellular regulators of IL-8 include IFN-γ, a cytokine notably present at low levels in newborns [24, 25]. Thus newborns with low levels of IFN-γ might be unable to down-regulate IL-8. Additionally, HIV *tat* has been shown to signal production of IL-8 [26], and ongoing genital tract viral replication during pregnancy could result in placental HIV *tat*. Prolonged exposure of fetal and neonatal T lymphocytes to elevated IL-8 likely leads to intracellular signaling of gene pathways associated with cell survival, proliferation, and metabolism [27]. Hypothetically, neutrophils and other granulocytes programmed to respond to IL-8 along a chemotactic gradient also might be unable to respond to the signals if there is no functional gradient present, disabling the intended effect of chemotaxis and instead resulting in an immune ‘confusion’ or ‘paralysis’ until levels of circulating IL-8/IL-6 return to homeostasis. The consequence of a lack of recruitment of neutrophils to the sites of tissue damage and infection is consistent with clinical descriptions of HEU morbidity and mortality.

Our analyses demonstrated a consistent pattern of significantly increased levels of chemotactic cytokines present in cord blood compared to delivery blood in HEU infants compared to HU infants (Fig. 2). The increased relative concentrations of IL-8, CCL3, CCL4, CCL20 in HEU circulation compared to maternal circulation suggests a coordinated response to inflammatory signaling. GM-CSF, although present at levels below that found in maternal circulation, is significantly increased in HEU compared to HU infants. IL-10 levels are increased in HEU infants, although still lower than levels found in maternal circulation. IL-10 is in part regulated by IL-6 levels, thus it is feasible higher circulating IL-6 levels release the inhibition on IL-10 production in HEU infants. Regardless the source of mediators detected in HEU cord blood, the finding of significantly elevated cytokines compared to both HIV unexposed control cord blood plasma as well as paired maternal blood collected at delivery suggests controlled HIV infection during pregnancy drives the fetal immune response.

This study had several strengths. Access to stored specimens from the pre-ARV era allowed us to eliminate the direct and indirect effect of ARVs on maternal and neonatal cytokine production. Longitudinal HIV testing within the parent cohorts allows us to rule out individuals with subsequent acquisition of HIV. While the inclusion of Kenyan HIV-uninfected mother-infant dyads is a strength of the study, allowing us to examine cytokine responses in populations of women living in similar socio-economic conditions, it raises a potential weakness considering the time difference in collection of samples from the two cohorts. Further research is needed to determine the cellular source of the elevated IL-8 and the mechanism of induction/or release from negative feedback for production of this cytokines. Additionally, because we selected normal birthweight and gestational age infants for both HEU and HU infants, we cannot assess the role of maternal and fetal systemic inflammation on birth anthropometry.

The majority of immune function studies of HEU compared to HU infants have evaluated cellular responsiveness following antigenic stimulation in vitro. Our studies focused on the prenatal cytokine environment in which presumably the cellular response would arise. In a study of plasma cytokines from HEU and HU infants born to HIV+ ARV-treated and healthy HIV− pregnant Brazilian women, HEU and HU cord blood plasma IL-8 levels were low and similar in HEU infants delivered via Caesarian section compared to HU infants delivered vaginally [28]. It remains an important public health goal to identify the effects of chronic maternal infections on fetal and infant immunity, correlations with infant health, and response to maternal antiviral treatments.

## Conclusions

We conclude that in utero stimuli stemming from infection generated an immune profile consistent with fetal/infant immune activation, particularly cytokines associated with macrophage activation, above what is expected at parturition. The weak association with maternal viral load raises the concern that HEU infants exposed to ARV may still be at risk for increased altered immune profile at birth.

## Abbreviations

AIDS: acquired immunodeficiency syndrome
ARV: antiretroviral
CBMC: cord blood mononuclear cells
CCL: chemokine (C–C motif) ligand
CXC: chemokine (C-X-C motif)
CX3CL: chemokine (C-X3-C motif) ligand
EDTA: ethylenediaminetetraacetic acid
GM-CSF: granulocyte macrophage colony stimulating factor
HEU: HIV exposed uninfected
HIV: human immunodeficiency virus
HU: HIV unexposed
IFNγ: interferon gamma
IL: interleukin
IP9: interferon gamma inducible protein 9
IQR: interquartile range
ITAC: interferon inducible T cell alpha chemoattractant
LMIC: low and middle income countries
PCR: polymerase chain reaction
PMTCT: prevention of mother to child transmission
SEM: structural equation modeling
STI: sexually transmitted infection
TMA: transcription mediated amplification
TNF: tumor necrosis factor
UNAIDS: Joint Unitied Nations programme on HIV/AIDS
